# Self-rated health as a predictor of mortality and healthcare use in older adults at high risk of hospitalisation: a prospective cohort study in Sweden

**DOI:** 10.1136/bmjopen-2024-091787

**Published:** 2025-09-01

**Authors:** Kristin Hansén, Johan Lyth, Anna Segernäs, Jenny Alwin, Magnus Nord

**Affiliations:** 1 Department of Health, Medicine and Caring Sciences, Linköping University, Linköping, Östergötland County, Sweden; 2 Primary Health Care Center Valla, Region Östergötland, Linkoping, Östergötland, Sweden; 3 Primary Health Care Center Ekholmen, Region Östergötland, Linkoping, Östergötland, Sweden

**Keywords:** Mortality, Frailty, GERIATRIC MEDICINE, Hospitalization, PUBLIC HEALTH

## Abstract

**Objective:**

This study aimed to evaluate the predictive value of self-rated health (SRH) on mortality and healthcare use in older adults (aged ≥75 years) at high risk of hospitalisation in comparison to an objective measure of comorbidities, the Charlson Comorbidity Index (CCI).

**Design:**

Prospective cohort study conducted within the research project ‘Proactive Primary Care for Frail Elderly Persons’.

**Setting:**

19 primary care practices in south-east Sweden, between January 2018 and December 2019.

**Participants:**

In total, 355 adults aged ≥75 years were included in the study. They were among the 11% older adults with the highest predicted risk of hospitalisation, as identified by a statistical prediction model for unplanned hospital admission.

**Outcome measures:**

Outcomes were all-cause mortality and healthcare use measured as hospital care days and the number of physician visits in primary and secondary care. These were analysed for different groups of SRH and comorbidities measured using the CCI.

**Results:**

SRH was grouped into Excellent/Very good, Good, Fair and Poor. The overall mortality rate was 26.5%. Compared with the *Poor* group, the adjusted HRs were significantly lower for *Excellent/Very good* (HR=0.2; 95% CI: 0.1 to 0.8, p=0.02) and *Fair* (HR=0.5; 95% CI: 0.3 to 1.0, p=0.04). Compared with the comorbidity group CCI 0–1, CCI 2–3 had an adjusted HR of 2.2 (95% CI: 1.1 to 4.6, p=0.03), CCI 4–5 had an adjusted HR of 2.6 (95% CI: 1.2 to 5.4, p=0.01) and CCI>5 had an HR of 4.9 (95% CI: 2.4 to 10.2, p<0.001). The number of hospital care days was 70% lower (adjusted relative difference=0.3; 95% CI: 0.1 to 0.8) for *Excellent/Very good* (3.9 days) compared with *Poor* (10.7 days). All groups of CCI diagnoses (2–3, 4–5 and >5) had significantly more hospital care days than CCI 0–1.

For physician visits in secondary care, both the SRH *Excellent/Very good* (p=0.004) and *Good* (p=0.02) groups had significantly fewer visits compared with *Poor*. In the comorbidity groups, no statistical differences were found between CCI categories.

**Conclusions:**

In a cohort of older adults at high risk of hospitalisation, the predictive value of SRH for risk stratification was limited. Objective health measures appeared to offer greater utility than SRH for guiding healthcare planning and tailoring interventions for vulnerable older adults in this cohort.

**Trial registration number:**

Clinical Trials NCT03180606.

**STRENGTHS AND LIMITATIONS OF THIS STUDY**

- Among the respondents, there were few missing data in the questionnaires.
- The data set was relatively small, partly explained by a high mortality in this vulnerable older population, but also a low response rate.
- Analysing perspectives on self-rated health and its association with frailty and risk assessments is of importance, especially for the most vulnerable older patient groups, for whom data are scarce.
- The study is based on reliable registry data regarding objective health measures, hospitalisation and mortality, as these were collected from a healthcare source covering all healthcare contacts of the population in the county of Östergötland.

## Introduction

 As the world’s population increases, the proportion of senior citizens is also growing. This demographic shift is a result of several factors, including reduced mortality rates among the young, lower birth rates and remarkable medical advancements.^1^ In response to this change in population structure, new and effective healthcare strategies are required.

A significant portion of the older population comprises robust and healthy individuals, even though the prevalence of multimorbidity increases with age.^2^ There is consensus to prioritise the most vulnerable older adults and those at the highest risk of hospitalisation. Hence, a diversified approach is needed to manage the varying needs within the elderly population.

Frailty has become a well-established concept for describing an individual’s susceptibility to functional decline and ability to recover from disease.^3^ There are numerous ways of describing and assessing frailty based on either of the main concepts of Linda Fried (Deficit model) or Kenneth Rockwood (Frailty Index).^3 4^ These frailty scales have demonstrated a high correlation with mortality.^3^,^5^

Although valuable, frailty instruments and assessments are sometimes criticised for their lack of specificity and for adding to the workload in primary care settings.^6^ Moreover, the predictive properties of different frailty assessment tools are not always consistent between studies, for example, in risk stratification for hospital admission.^7 8^ This highlights the importance of continuously refining and developing tools to more effectively address the needs of the older population and identify individuals at risk. Various instruments have been tested for screening to evaluate the risk of hospital admission, functional decline or frailty.^9^,^11^ Assessing the risk of hospital admission in older adults using administrative data enables resource allocation and interventions for those most in need and may be cost-effective.^11^ Incorporating non-medical data, such as gait speed or self-rated health (SRH), can further improve the ability of prediction models in identifying older adults at high risk of hospital admission.^7 12^

SRH is gaining recognition as a useful and convenient tool in general practice and risk assessment of adverse health outcomes and mortality. Due to its simplicity and ability to cover various dimensions within a single question, ‘how is your health?’, and together with traditional risk factors, such as diabetes, smoking, etc, it can aid as a comprehensive screening tool to assess a person’s health status.^13^ Numerous epidemiological studies have demonstrated its predictive properties for mortality.^14 15^ This one-question assessment, most often rated on a scale from *‘Excellent*’ to *‘Bad*’, has gained considerable attention as an additional dimension to health surveys and has been proposed as a method to identify groups at risk for targeted interventions.^14 16^

Furthermore, SRH has been shown to be associated with objective health measures, such as the Charlson Comorbidity Index (CCI). A decline in health status is associated with a decline in SRH, but the relationship is non-linear. In response to new chronic conditions, older individuals with greater pre-existing comorbidity are less likely to decrease their health assessments than younger, healthier individuals.^17^

CCI is based on the ICD-10 diagnosis for 19 different medical conditions, with each contributing 0–6 points to the total score. It is a weighted index for predicting the risk of 1-year mortality after hospitalisation with comorbidities.^18^ The CCI is based on physical conditions, unlike, for example, frailty scales, which often include functional and mental aspects.^19^ In contrast to comorbidity, the term ‘multimorbidity’ can be used to cover broader aspects beyond medical conditions, including general physical and social functional status, geriatric problems and mental health. In this text, comorbidities refer to coexisting chronic diagnoses, while multimorbidity emphasises the complex interactions of coexisting diseases, including the resulting loss of function.^20^

Previous studies have explored SRH and found it to be acceptable and feasible as an adjunct to objective medical measures.^21^ The correlation between SRH and mortality remains robust, even for specific conditions, such as cancer, heart disease and for patients with unplanned hospital admission.^22 23^ However, subjective health assessments may not always align with objective health measures, and positive self-reported well-being can coexist with multimorbidity and frailty. Older adults in general, even with coexisting chronic conditions, seem to rate their health better than expected when compared with objective health measures.^24^ Some studies have stated that multimorbidity, rather than comorbidities alone, contributes significantly to perceptions of health.^20^

While SRH appears to predict mortality, it remains unclear whether this correlation persists among older adults with comorbidities, a population at high risk of frailty. Subsequently, the question remains whether SRH can be used to improve risk assessment in a population with a high prevalence of frailty and multimorbidity.

### Aim

The present study aim to explore the predictive value of SRH on mortality and healthcare use in a group of older adults (aged ≥75 years) at high risk of hospitalisation, and to compare it to the predictive value of an objective health measure, the CCI.

## Method

### Study design and setting

This is a prospective cohort study conducted within the research project ‘Proactive Primary Care for Frail Elderly Persons’.^25^ The original study, a pragmatic controlled trial, examined the effects of comprehensive geriatric assessment (CGA) adapted to primary care in a group of older adults (aged ≥75 years) at high risk of hospitalisation. Participants were identified using a statistical prediction model. Emergency-room visits, age, number of non-physician visits and number of physician visits were the most important variables for the model.^26 27^

The intervention trial took place at nine primary care practices in a region in south-east Sweden. 10 control practices were matched to the intervention practices with respect to the number of registered older adults and geographic location. The control practices provided usual care.

Sweden’s healthcare system is tax-funded and provides universal access to care for all residents, with services primarily managed by regional authorities. Different primary care practices are comparable in terms of care.

### Participants

The participants in the intervention trial were selected in March 2017 using a statistical prediction model that calculated the risk score for hospital admission in the coming 12 months, based on routine healthcare data from the preceding year. In total, 1308 participants were included in the trial. The risk score range is 0–1, and a higher score reflects a higher risk of hospitalisation.^27^ The most important variables were healthcare use and age. The follow-up period lasted 24 months (from January 2018 to December 2019).

The participants represented the 11% of the total population aged over 75 years at the 19 practices with the highest risk scores, consisting of respondents from both the intervention and control groups. This study includes participants who responded to a baseline questionnaire and consented to analysis of their healthcare use.

A postal questionnaire was mailed to all participants on three occasions during the study: at baseline, at 10 months of follow-up and at 22 months of follow-up. In connection with the baseline questionnaire, participants were asked for their consent to analyse their answers along with their healthcare use. Participants were asked to complete the questionnaire themselves (with assistance from a family member or caregiver if needed). The questions covered background information (eg, age and socioeconomic factors), need for assistance in daily life, loneliness, mental aspects and pain and health-related quality of life (HRQoL) measured with the EQ-5D-3L (EQ-5D descriptive system three levels) and the EQ-VAS (EQ visual analogue scale).^11^

### Outcome measures

SRH was collected from the baseline postal questionnaire based on the answers to the primary SRH question: ‘In *general, would you say your health is: Excellent, Very good, Good, Fair or Poor?*’. Comorbidity was measured using the CCI,^18^ with scores grouped as 0–1, 2–3, 4–5 and >5.

Mortality was quantified in terms of the number of days survived by each individual during the follow-up period that lasted 24 months (from January 2018 to December 2019).

Healthcare use was quantified as the number of hospital care days and the number of visits to primary and secondary care physicians. Data were imported from the County Council of Östergötland’s computerised information system, which stores statistics for all healthcare in the county (Care Data Warehouse, described in the previous article).^26^ Information on diagnoses for the CCI was collected from the same source.

### Statistical analysis

The data were analysed using R V.4.4.3 and IBM SPSS Statistics V.29.0.0.0. Descriptive statistics at baseline were used to summarise the characteristics of each SRH group. As few respondents (n=2) rated their health as excellent, groups were combined for further analysis into four categories: *Excellent/Very good*, *Good, Fair* and *Poor*. Differences in categorical variables were analysed using Fisher’s exact test and continuous variables using one-way ANOVA (Analysis of Variance). Cumulative mortality was performed using Kaplan-Meier, and Cox regression was used to calculate HRs for mortality in the different groups of SRH and CCI, respectively. The number of hospital care days and physician visits was analysed with negative binomial regression, with groups based on SRH and CCI evaluated separately. Negative binomial regression is more suitable for count data than linear regression, as the distribution of counts is often skewed with a higher proportion of low values. All analyses were adjusted for age, sex and level of education. We also performed sensitivity analyses, excluding patients diagnosed with dementia.

### Patient and public involvement

The research project ‘Proactive Primary Care for Frail Elderly Persons’ has been discussed and presented widely to different stakeholders, including patients, healthcare managers and healthcare policymakers. The specific results from this study have also been communicated to key personnel in clinical practice.

## Results

**Figure 1:**
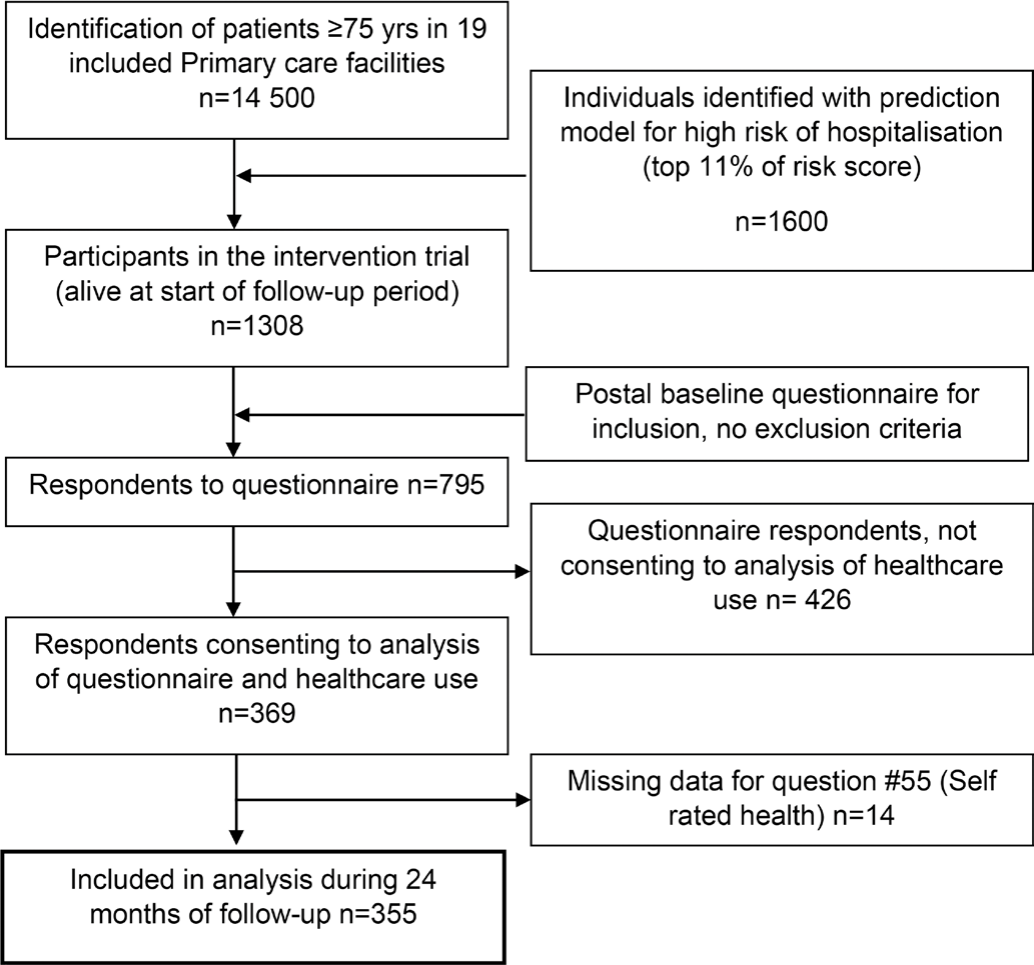
Participant flow chart.

In total, 355 respondents had responded to the primary SRH question and consented to the analyses of their answers along with their healthcare use (figure 1).

### Demographics

Participants had a mean age of 83.9 years (SD 5.4), and a significantly larger proportion were men (56.9%). At the end of the 2-year follow-up, 94 individuals were deceased, resulting in a mortality rate of 26.5%. The mean risk score for hospital admission was 0.34 (SD 0.18), with the lowest mean risk score for SRH Excellent/Very good (0.28 (SD 0.08)) and the highest for SRH Poor (0.38 (SD 0.22)). The differences in mean risk score were non-significant. The four groups of SRH had significant differences (p<0.001) in EQ-VAS scores, with the highest average rating in SRH Excellent/Very good (81.8 (SD 15.0)) and the lowest rating in SRH Poor (34.0 (SD 18.8)) (table 1).

**Table 1:** Characteristics of study participants, grouped by SRH

	Total	Excellent/ very good	Good	Fair	Poor	P value
Number of participants (%)	355	28 (7.9)	90 (25.4)	188 (53.0)	49 (13.8)	
Age, mean (SD)	83.9 (5.4)	82.3 (5.4)	84.5 (5.5)	83.9 (5.3)	84.0 (5.2)	0.31
Women (%)	153 (43.1)	9 (32.1)	24 (26.7)	95 (50.5)	25 (51.0)	<0.001
Men (%)	202 (56.9)	19 (67.9)	66 (73.3)	93 (49.5)	24 (49.0)	
Level of education, n (%)						0.09
No education	10 (2.8)	0 (0)	3 (3.3)	2 (1.1)	5 (10.2)	
Primary and secondary school	156 (43.9)	10 (35.7)	40 (44.4)	83 (44.1)	23 (46.9)	
High school	117 (33.0)	13 (46.4)	25 (27.8)	69 (36.7)	10 (20.4)	
Higher education (college/university)	64 (18.0)	4 (14.3)	20 (22.2)	30 (16.0)	10 (20.4)	
Missing	8	1	2	4	1	
Total	355 (100)	28 (100)	90 (100)	188 (100)	49 (100)	
Accommodation (%)						0.05
Ordinary housing—independent	259 (73.0)	27 (96.4)	67 (74.4)	136 (72.3)	29 (59.2)	
Ordinary housing—home help	71 (20.0)	1 (3.6)	19 (21.1)	37 (19.7)	14 (28.6)	
Nursing home	10 (2.8)	0 (0)	1 (1.1)	7 (3.7)	2 (4.1)	
Missing	15	0	3	8	4	
Total	355 (100)	28 (100)	90 (100)	188 (100)	49 (100)	
Cohabitation status (%)						0.50
Living alone	161 (45.4)	10 (35.7)	39 (43.3)	88 (46.8)	24 (49.0)	
Living with partner	186 (52.4)	18 (64.3)	50 (55.6)	96 (51.1)	22 (44.9)	
Living with children	5 (1.4)	0 (0)	1 (1.1)	2 (1.1)	2 (4.1)	
Missing	3	0	0	2	1	
Total	355 (100)	28 (100)	90 (100)	188 (100)	49 (100)	
EQ-VAS (n=330), mean (SD**)**	54.4 (20.9)	81.8 (15.0)	63.2 (18.3)	51.6 (16.7)	34.0 (18.8)	<0.001
Risk score, mean (SD)	0.34 (0.18)	0.28 (0.08)	0.34 (0.18)	0.34 (0.19)	0.38 (0.22)	0.13

As a sensitivity analysis, Kruskal-Wallis tests were performed for the variables age (p=0.27), risk score (p=0.72) and EQ-VAS (EQ-Visual Analogue Scale) (p<0.001).

SRH, self-rated health.

### Survival analysis

**Figure 2:**
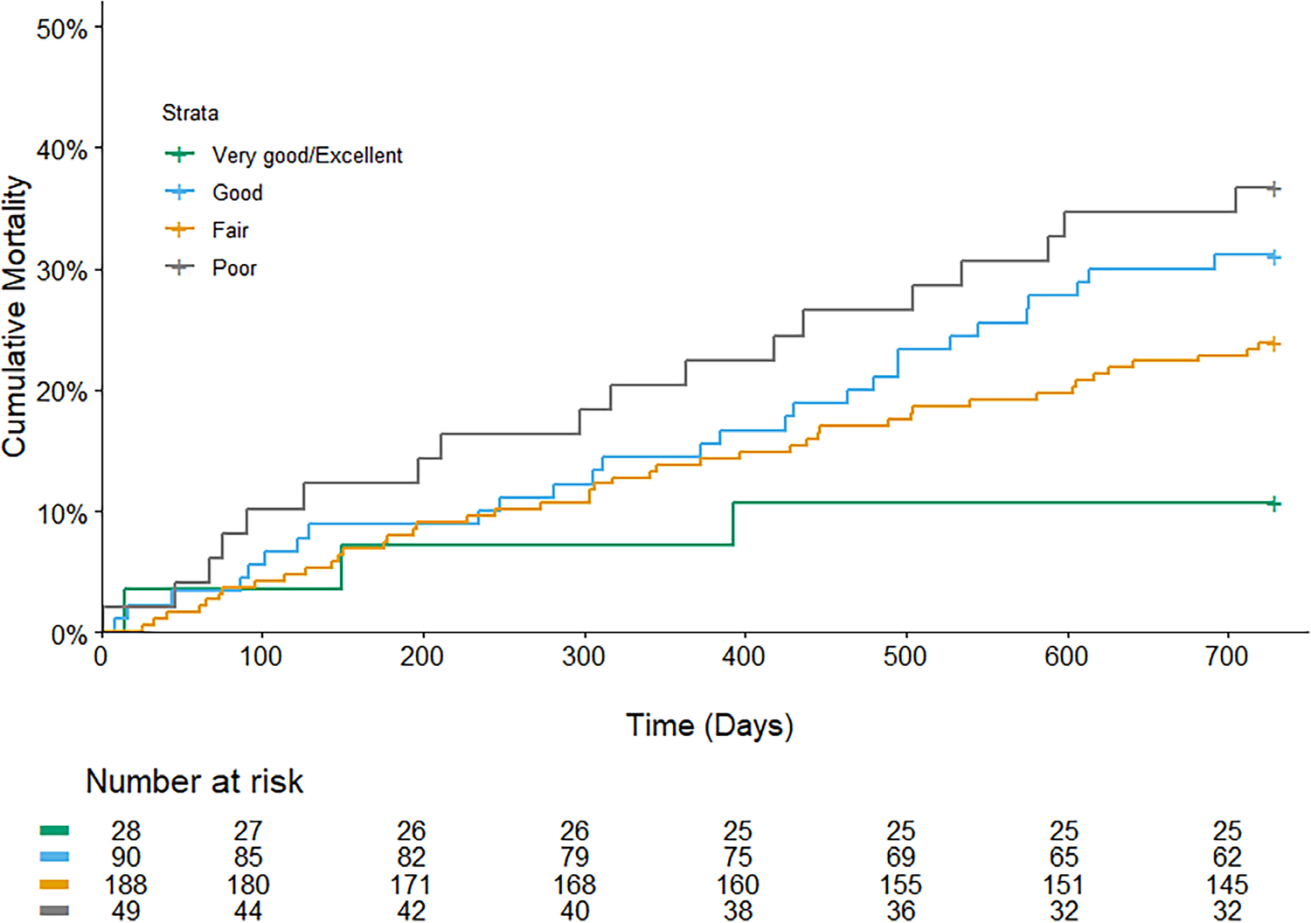
Mortality during 2 years of follow-up, comparing different groups of self-rated health.

**Figure 3:**
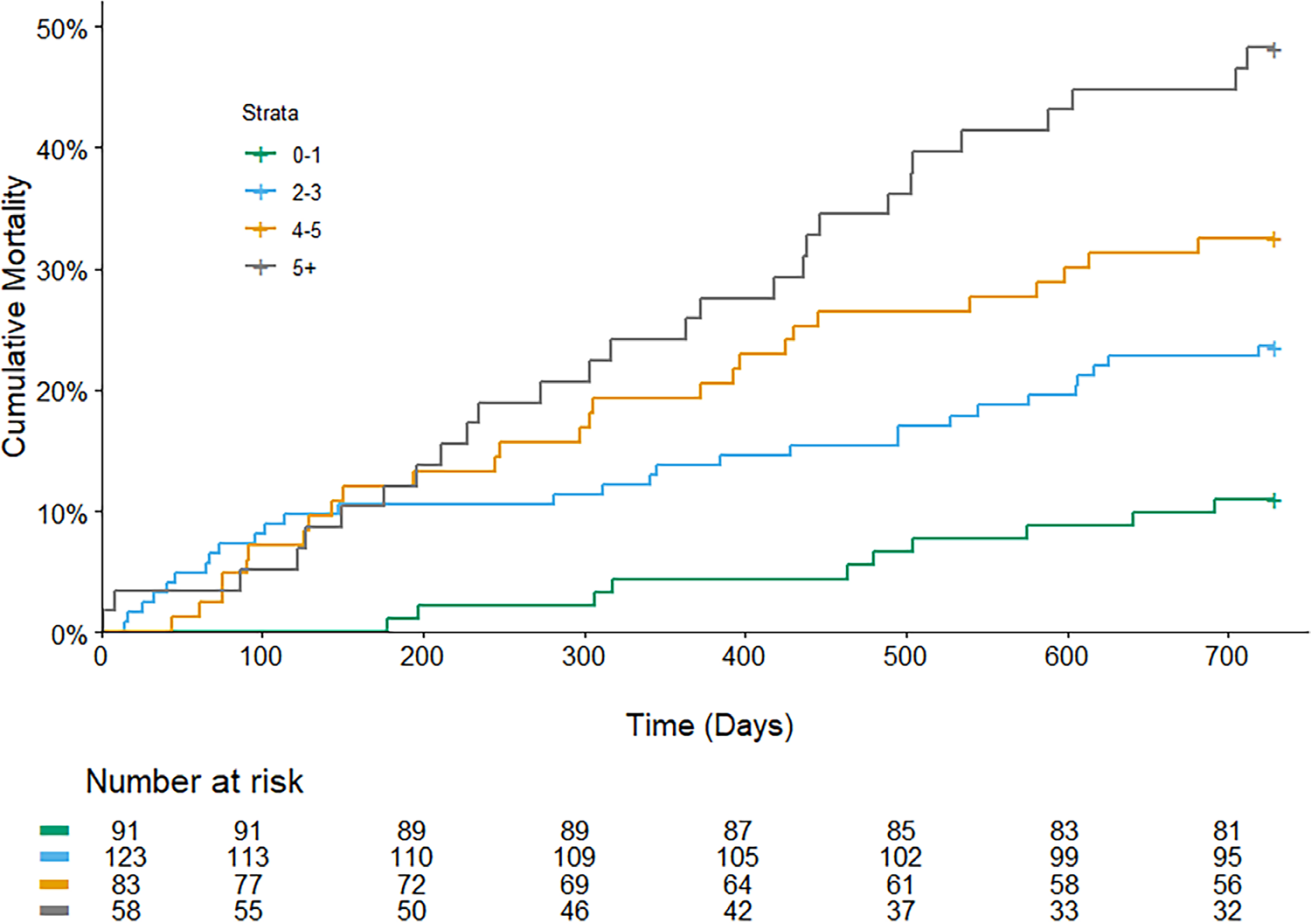
Mortality during 2 years of follow-up, comparing different groups of Charlson Comorbidity Index.

The Kaplan-Meier survival curves for the four groups of SRH are shown in figure 2. The adjusted HR for the *Excellent/Very good* (HR=0.2; 95% CI: 0.1 to 0.8, p=0.02*)* and *Fair* (HR=0.5; 95% CI: 0.3 to 1.0, p=0.04*)* groups were significantly lower compared with the *Poor* group (table 2). The sensitivity analysis, excluding patients with dementia, resulted in a significant effect on *Excellent/Very good*, but *Fair* was no longer significant compared with *Poor*. A Kaplan-Meier survival analysis for the different CCI scores is shown in figure 3. Compared with CCI 0–1, CCI 2–3 had an adjusted HR of 2.2 (95% CI: 1.1 to 4.6, p=0.03), CCI 4–5 had an adjusted HR of 2.6 (95% CI: 1.2 to 5.4, p=0.01), while the HR for CCI>5 was 4.9 (95% CI: 2.4 to 10.2, p<0.001).

**Table 2:** Cox analyses of mortality for study participants, grouped by SRH and CCI

	Crude HR (95% CI)	P value	Adjusted HR (95% CI)	P value
SRH				
Poor	1 (reference)		1 (reference)	
Fair	0.6 (0.3 to 1.0)	0.06	0.5 (0.3 to 1.0)	0.04
Good	0.8 (0.4 to 1.4)	0.46	0.6 (0.3 to 1.1)	0.10
Excellent/very good	0.3 (0.1 to 0.9)	0.03	0.2 (0.1 to 0.8)	0.02
CCI				
0–1	1 (reference)		1 (reference)	
2–3	2.4 (1.2 to 4.9)	0.02	2.2 (1.1 to 4.6)	0.03
4–5	3.5 (1.7 to 7.2)	<0.001	2.6 (1.2 to 5.4)	0.01
>5	5.6 (2.7 to 11.5)	<0.001	4.9 (2.4 to 10.2)	<0.001

All models were adjusted for age, sex and level of education.

.CCI, Charlson Comorbidity Index; SRH, self-rated health.

### Hospital care days

The number of hospital care days was 70% (adjusted relative difference=0.3; 95% CI: 0.1 to 0.8) lower for the *Excellent/Very good* (3.9 days) group compared with the *Poor* (10.7 days) group (table 3). In the sensitivity analysis, excluding patients with dementia, the mean hospital care days were 3.0 for the *Excellent/Very good* group, and this remained the only significant (p≤0.001) effect compared with the *Poor* group.

**Table 3:** The number of hospital care days and physician visits in primary and secondary care, by SRH and CCI

	Hospital care days		Primary care		Secondary care	
	Mean (SD)	Adjusted relative difference (95% CI)	Mean (SD)	Adjusted relative difference (95% CI)	Mean (SD)	Adjusted relative difference (95% CI)
Poor	10.7 (13.6)	Reference	5.4 (5.2)	Reference	7.8 (8.3)	Reference
Fair	8.6 (13.6)	0.9 (0.5 to 1.5)	5.2 (4.3)	1.0 (0.7 to 1.3)	6.6 (7.8)	0.8 (0.6 to 1.1)
Good	9.2 (15.1)	0.8 (0.4 to 1.6)	4.8 (4.0)	0.9 (0.7 to 1.3)	5.2 (6.3)	**0.6 (0.4 to 0.9**)
Very good/excellent	3.9 (7.0)	**0.3 (0.1 to 0.8**)	4.3 (3.8)	0.8 (0.6 to 1.3)	4.1 (3.8)	**0.5 (0.3 to 0.8**)
CCI						
0–1	3.1 (7.0)	Reference	5.4 (5.2)	Reference	5.7 (7.6)	Reference
2–3	8.2 (13.1)	**2.4 (1.5 to 4.0**)	5.0 (3.9)	0.9 (0.7 to 1.2)	6.2 (7.2)	1.1 (0.8 to 1.4)
4–5	12.3 (17.0)	**3.2 (1.9 to 5.7**)	5.4 (4.2)	1.1 (0.8 to 1.4)	5.5 (6.9)	1.0 (0.7 to 1.4)
>5	13.1 (14.4)	**3.8 (2.1 to 7.1**)	4.2 (3.8)	0.8 (0.6 to 1.1)	8.1 (7.6)	1.4 (1.0 to 2.1)

Bold indicates statistical significance.

Mean values are unadjusted.

Relative differences were adjusted for age, sex and level of education.

CCI, Charlson Comorbidity Index; SRH, self-rated health.

For comparison, the mean values of hospital care days were analysed for the different groups of CCI score, as shown in table 3. All groups differed significantly from CCI 0–1.

### Physician office visits

The number of primary care and secondary care (outpatient) visits was analysed for the four SRH groups. There were no significant differences between SRH categories in primary care visits over the 2 years of follow-up. For secondary care, both *Excellent/Very good* (p=0.004) and *Good* (p=0.02) groups had significantly fewer visits compared with the *Poor* group. The sensitivity analyses, excluding patients with dementia, resulted in p values of 0.002 for the *Excellent/Very good* group and p values of 0.007 for the *Good group* compared with the *Poor* group.

There were no significant differences between CCI categories in primary care or secondary care visits over the 2 years of follow-up.

## Discussion

The present study aimed to examine the predictive value of SRH and comorbidities (CCI) on mortality and healthcare use in a group of older adults (aged ≥75 years) at high risk of hospitalisation.

In this cohort, SRH showed a trend towards predicting mortality or hospital care days when comparing the best ratings of health with the worst ratings; however, intermediate ratings demonstrated less discriminatory abilities.

There were small, but significant differences for mortality comparing the groups of *Fair* and *Excellent/Very good* to *Poor*, when adjusted for age, sex and level of education. An indication of increased healthcare use, in terms of secondary care visits, was observed in the SRH *Excellent/Very good* and *Good groups* compared with the *Poor group* when adjusted for age, sex and level of education. Hospital care days were significantly lower in the SRH group of *Excellent/Very good* compared with the *Poor* group; however, no other significant differences were observed. The number of visits to primary care physicians did not differ significantly between the SRH groups.

The CCI was used as the objective health measure and had a significant correlation with mortality and visits in secondary care. A CCI score of >5 showed an almost sixfold risk of mortality compared with the CCI 0–1 group. All groups with a CCI score >2 had a significantly higher mean of hospital care days compared with the CCI 0–1 group. The more comorbidities, the higher the risk of mortality, hospital care days and visits in secondary care. This was expected given that CCI is a validated risk prediction tool.^18^ However, no significant differences were shown for CCI in primary care visits.

Participants in this cohort had generally poorer SRH compared with similar groups in previous studies.^28 29^ The population had more comorbidities, as measured by CCI, and an increased overall mortality compared with the findings in other studies investigating the link between SRH and mortality in older age groups.^29^ The overall mortality rate was 26.5% during the 2-year follow-up compared with 14.2% in the general population aged >75 years in the county of Östergötland in the same period.^30^ This was expected, as the cohort was part of the population with the highest risk of hospital admission, as calculated by the prediction model.^11^ The prediction model was validated in a separate prospective study and demonstrated good predictive properties for cumulative mortality and hospitalisation over 2 years. The groups with the highest risk scores had considerably higher 2-year cumulative mortality, 43% in the 95–100 percentile compared with 7% in the lowest risk score percentile 0–60, and an increasing number of hospital care days with increasing risk score.^31^ There were no significant differences between the SRH groups in terms of risk score (table 1), which indicates that individuals at higher risk do not necessarily rate their health as poorer.

Previous studies have found that the correlation between SRH and mortality exists in older age, although it may decline or not be as strong as for objective health measures.^21 32^ The results of this study are consistent with previous findings that SRH assessments differ more from objective health measurements within older populations.^33^ In the oldest old, the group where SRH has shown a weaker correlation with objective health measures, limitations in functional ability are positively correlated to poorer health ratings.^34^ In patients with dementia, the associations between objective indicators and SRH are weakened, except for fatigue, dizziness and vision impairment.^35 36^ Factors associated with good SRH not only include lower levels of multimorbidity but also physical and functional resilience.^37^ These findings from previous research indicate that functional decline has a greater impact on perceived health in this age group.^34 35^ Furthermore, physical health deteriorates more than mental health with increasing multimorbidity.^38^ This could be explained by the process of psychological and social adaptation to the gradual loss of function and increasing illness that many experience as they age.^36^

As mentioned, this cohort constitutes the older part of the population with the highest use of healthcare. Adaptation to poor physical health in this group could be explained by a recalibration response shift over time; that is, standards for what is considered ‘good health’ are lowered over time and affected by previously experienced illnesses.^36^ This may explain why SRH does not exhibit the same discriminatory abilities as in other cohorts, as factors other than physical health itself might influence SRH.

Notably, EQ-VAS (see table 1) correlated well with SRH in this cohort; that is, worse SRH also had lower scores on EQ-VAS, which can be considered to validate that the self-rating of health correlated well with the HRQoL.

This cohort study did not exclude participants with cognitive impairment. Dementia and cognitive impairment are more prevalent in older age and are associated with multimorbidity and cardiovascular disease. In this study population, we expected a higher prevalence of cognitive impairment and dementia compared with the general population aged over 75 years. The cohort can be considered representative of the population aged ≥75 years, a population with a high risk of hospitalisation in the county.

Including a health rating is valuable, particularly when studying populations that include individuals with cognitive impairment or dementia, as these groups are often under-represented.

To our knowledge, this is the first study to examine the predictive value of SRH in a population of older people at high risk of hospitalisation. Previous studies have been based on general age cohorts, emergency department data on hospital admission or populations with a specific condition (e.g., heart failure).^22 23^ Existing research in the field suggests that SRH has predictive value comparable to other predictive instruments using administrative data.^32 39^ Our findings could not confirm this. We believe that objective measures, such as the CCI, are of greater value than SRH as an additional parameter when planning healthcare and tailoring interventions for prefrail and frail groups of older adults. Incorporating SRH into models for identifying persons at high risk of hospitalisation or mortality could potentially increase bias.^24^ However, introducing SRH in primary care consultations may contribute to a holistic approach and help understand how individuals think about their health and potentially reduce unnecessary referrals to secondary care. Further research in this area is needed to better understand the implications and refine the role of SRH in these contexts.

### Study strengths and limitations

This study is based on reliable registry data on objective health measures, hospitalisation, and mortality, as these were collected from a healthcare source covering all healthcare contacts of the population in the county of Östergötland. Furthermore, there were a few missing data on the primary SRH question and background information of the respondents.

The five categories of SRH had to be grouped together to compensate for the small groups in the higher ratings of SRH. This is consistent with the methodology of similar studies, as the groups of higher ratings tend to be smaller and rarely show significant differences in comparing analyses in the range from Good to Excellent.^15 32 34^ Including individuals with cognitive impairment and dementia may affect the results, but the sensitivity analysis excluding patients with dementia resulted in only minor differences and did not affect our interpretation of the results. Additionally, as mentioned above, we consider the representation of these individuals important when studying the correlation of SRH with mortality and healthcare use in the population aged ≥75 years at high risk of hospitalisation.

The data set was relatively small compared with epidemiological studies on SRH and correlation with mortality and hospitalisation. This affects the generalisability of the results. However, our cohort was larger or of similar size compared with questionnaire-based studies on vulnerable adults and, therefore, added value to the research field.^21^

Only 27% of the eligible participants from the original trial could be included in this study. Compared with the intervention trial, participants in this study had the same mean age and risk of hospitalisation but a higher percentage of men (57% vs 49%).^40^ Men were mainly over-represented in the SRH group Good, which we have adjusted for in our analyses. The results are considered to be generalisable between countries with similar socioeconomic status and healthcare systems. Self-ratings of health can also be affected by culture and socioeconomic status. To generalise the findings globally, it is necessary to reproduce this study in wider settings and other types of healthcare systems.

## Conclusions

In a cohort of older adults at high risk of hospitalisation, there was a trend towards a graded relationship between SRH and mortality, but we consider that the value of SRH for risk stratification was limited. The objective health measure CCI had a stronger and graded correlation with mortality and hospital care days.

For further development of an accurate predictive model to target individuals in the greatest need of an intervention, such as CGA, we propose not to add SRH. The results suggest that objective measures, such as CCI, hold greater value than SRH for guiding healthcare planning and tailoring interventions for older, vulnerable groups.

Further studies are required to explore the integration of an individualised perspective into existing frailty and risk assessment instruments. SRH is well correlated to perceived quality of life. Addressing reflections and thoughts of health is essential to enhance trust and may be a way to increase self-efficacy and possibly reduce healthcare needs in older adults with multimorbidity and vulnerability.

## Data Availability

Data are available upon reasonable request.
