# Supplementary material for: Self-rated health as a predictor of mortality and healthcare use in older adults at high risk of hospitalisation: a prospective cohort study in Sweden
Source: BMJ Open. 2025 Sep 1;15(9):e091787. doi: 10.1136/bmjopen-2024-091787 (PMC12406932; doi:10.1136/bmjopen-2024-091787)
Supplement: Supplementary file 1 [file bmjopen-2024-091787.reviewer_comments.pdf]

PEER REVIEW HISTORY

BMJ Open publishes all reviews undertaken for accepted manuscripts. Reviewers are asked to complete a checklist review form and are provided with free text boxes to elaborate on their assessment. These free text comments are reproduced below.

ARTICLE DETAILS

Title (Provisional)

Self-rated Health as a Predictor for Mortality and Healthcare Use in Older Adults at High Risk of Hospitalisation – a Prospective Cohort Study in Sweden

Authors

Hansén, Kristin; Lyth, Johan; Segernäs, Anna; Alwin, Jenny; Nord, Magnus

---

VERSION 1 - REVIEW

**Reviewer**	**1**
**Name**	**Zaitsu, Masayoshi**
**Affiliation**	**University of Occupational and Environmental Health Japan, Center for Research of the Aging Workforce**
**Date**	**19-Oct-2024**
**COI**	**None**

General comment:

This cohort study investigates the predictive value of self-rated health (SRH) for morbidity and mortality in older adults at high risk of hospitalization. While the topic is important and relevant for healthcare resource allocation, there are several significant issues that need to be addressed. The study's reliance on a questionnaire-based approach raises concerns about data reliability, particularly regarding cognitive decline and the presence of comorbidities.

Major comments

A significant issue is the low response rate, which likely introduced substantial sampling bias. This could affect the generalizability and reliability of the findings.

Cognitive decline, especially among participants with dementia, may affect the accuracy of SRH responses, making it difficult to draw robust conclusions. At a minimum, a sensitivity analysis excluding participants with dementia should be performed to assess how this factor may have impacted the results.

The manuscript lacks clear definitions of both the exposure and outcome variables. While the primary aim is to examine the association between SRH and mortality, there is also mention of a relationship between the Charlson Comorbidity Index (CCI) and mortality. The

rationale for comparing SRH and CCI in predicting mortality needs to be better integrated into the hypothesis and clearly explained within the study's framework.

Minor comments:

1. Are there any major differences between Sweden and other countries in healthcare resource usage that could affect the generalizability of the findings?

2. The term "EQ-VAS" should be fully spelled out the first time it is introduced.

3. Is it appropriate to group participants by SRH only once at the beginning of the study? Consider discussing potential changes in individual SRH responses during the two-year follow-up, particularly in this vulnerable population.

4. The data on hospital care days (p.7, lines 49–50) and physician office visits (p.7, line 57–p.8, line 10) would be clearer if presented in a table.

5. For Figures 2 and 3, please provide the "Number at risk." Also, consider including a table with hazard ratios (HRs) where appropriate.

6. On p.9, line 15, the finding that there is little correlation between SRH and mortality or hospital care days in older adults at high risk of hospitalization is a key result. However, the discussion lacks depth and relies too much on speculation based on previous studies. The manuscript should provide a more thorough explanation of why this finding emerged.

7. On p.9, line 57, the manuscript suggests there is no reason to expect other items in the questionnaire to significantly affect SRH perception. However, this statement requires further justification or supporting evidence.

**Reviewer**	**2**
**Name**	**Kelson, Zoe**
**Affiliation**	**University of Exeter, Mathematics**
**Date**	**19-May-2025**
**COI**	**None**

This study evaluated the predictive value of self-rated health (SRH) and the impact of comorbidities on morbidity and mortality in older adults (aged 75+) at high risk of hospitalisation.

Reviewer comments:

"There was no significant difference between the three groups of SRH in terms of mortality or hospital care days. However, groups with higher CCI had significantly higher mortality and a greater number of hospital care days." [Abstract]

Can statistical outcomes please be reported alongside study results in the Abstract?

"The original trial included individuals from 19 primary care practices in Sweden, aged ≥75 years and at high risk of hospitalisation selected using a prediction model. A total of 1308 participants received a baseline questionnaire; 355 individuals were included." [Abstract]

and

"A total of 1308 participants were selected in March 2017 using a statistical prediction model that calculates a risk score for hospital admission in the coming 12 months using routine health care data from the preceding year...

The selected participants represented the 11% with highest risk score of the total population over 75 years at the 19 practices."

and

"The questionnaire was provided to a total number of 1308 participants. The survey was completed by 798 individuals, out of which 355 respondents consented to further analysis and had responded to the primary SRH-question"

Can the authors please confirm if the included cohort can be considered to be representative for generalisability of the study findings?

Can differences by primary care practice please be explored?

"The questions covered background information (e.g., age, socioeconomic factors); need for assistance in daily life; loneliness; mental aspects; pain and health related quality of life (HRQoL) measured with the EQ-5D-3L (EQ-5D descriptive system three levels) and the EQ-VAS"

and

"Data were imported from the County Council of Östergötland's computerised information system, where statistics for all healthcare in the county are stored (Care data warehouse, described in previous article)."

Can the authors please confirm if missing data (with covariate missing data shown in Table 1, and outcome missing data shown in Figure 1) can be considered to be missing at random?

How was covariate missing data handled in the analyses and models?

"Self-rated health was assessed by the question:" In general, would you say your health is: Excellent, Very good, Good, Fair or Poor". The responses were divided into three groups: Good, Fair and Poor. "

and

"As few respondents rated their health as Excellent or Very good, groups were combined for further analysis into three categories: Good (including Excellent, Very good and Good); Fair (fair) and Poor (poor)."

Can the outcome please also be analysed as five groups in an exploratory sensitivity analysis?

"Differences in categorical variables were analysed using Chi-square test and continuous variables using one-way ANOVA. Cumulative mortality was performed using Kaplan-Meier and Cox regression was used to calculate hazard ratios (HRs) for mortality in the different groups of SRH and CCI, respectively. Comparisons of mean values of hospital care days and Physician visits were analysed with a one-way ANOVA grouped by SRH and CCI, separately. In case of a significance, a Tukey post hoc test was conducted."

Can the authors please confirm if and how they assessed normality for the application of parametric tests (e.g. ANOVA)?

Can the authors please clarify if the Cox regression analyses adjusted for confounders? Can covariates please be specified here in the methods?

"the majority were men (56.9%), with no statistical difference between the groups of SRH"

Can the authors please double check the wording in the Results text, as Table 1 shows there is a statistical difference in sex ratio between the three groups ($p<0.001$)?

"The three groups of SRH had significant differences ($p\leq0.001$) in EQ-VAS scores, with the highest average rating of EQ-VAS in SRH Good 67.7 (SD 19.2), followed by SRH Fair 51.6 (SD 16.7) and SRH Poor 34.0 (18.8). (Table 1) "

and

"Table 1. Characteristics of study participants, grouped by SRH"

Can statistically significant differences in sex, education and EQ-VAS score (shown in Table 1) please be commented on in the Results text, and adjusted for in the analyses?

Figure 2:

Can the dichotomisation of 'Poor' versus 'not-Poor' (i.e. 'Fair' and 'Good' combined) SRH outcome please be analysed? It might be that the cohort analysed in this study is better at differentiating between 'Poor' and 'Fair and above' health statuses, than using more granular categories such as 'fair', 'good', 'very good' and 'excellent'?

"The data set was relatively small compared to studies on SRH and correlation to mortality and hospitalisation, based on epidemiological data. However, our cohort was larger or of similar size compared to questionnaire-based studies on vulnerable adults, and therefore add value to the research field.23 The five categories of SRH had to be grouped together to compensate for small groups of the higher ratings of SRH. This is consistent with the methodology of similar studies, as the groups of higher ratings tend to be smaller and rarely shows significant differences in comparing analyses in the range from Good to Excellent.17, 34 As the SRH question is embedded in a larger questionnaire, other items might affect the individuals' perception of their health, i.e., question order effects. We did not investigate this

aspect further in the present study, but we have no reason to believe that it would have a significant impact on the results."

A discussion on the study limitations has been provided.

Thanks for providing a copy of the STROBE checklist.

VERSION 1 - AUTHOR RESPONSE

Reviewer: 1
Prof. Masayoshi Zaitsu, University of Occupational and Environmental Health Japan
Comments to the Author:

General comment:
This cohort study investigates the predictive value of self-rated health (SRH) for morbidity and mortality in older adults at high risk of hospitalization. While the topic is important and relevant for healthcare resource allocation, there are several significant issues that need to be addressed. The study's reliance on a questionnaire-based approach raises concerns about data reliability, particularly regarding cognitive decline and the presence of comorbidities.

Major comments
A significant issue is the low response rate, which likely introduced substantial sampling bias. This could affect the generalizability and reliability of the findings. Cognitive decline, especially among participants with dementia, may affect the accuracy of SRH responses, making it difficult to draw robust conclusions. At a minimum, a sensitivity analysis excluding participants with dementia should be performed to assess how this factor may have impacted the results.

Response: Thank you for your concern. An important principle for us when conducting the study has been to obtain as broad and fair representation of the study population as possible. Mild cognitive impairment is common in this study population and data from these patients are also of importance to fully describe the self-rated health of this population. The objective was to study SRH from the patient perspective and compare it with an objective health measure such as Charlson Comorbidity score to explore differences in these measures. Sensitivity analysis has been carried out where individuals with diagnosis dementia was excluded, with no major impact on the results. This has been added in the results section and in the discussion line 330-335 and 364-369.

The manuscript lacks clear definitions of both the exposure and outcome variables. While the primary aim is to examine the association between SRH and mortality, there is also mention of a relationship between the Charlson Comorbidity Index (CCI) and mortality. The rationale for comparing SRH and CCI in predicting mortality needs to be better integrated into the hypothesis and clearly explained within the study's framework.

Response: Thank you for this comment. This has now been addressed and elaborated in the manuscript, p 4, line 116-129.

Minor comments:

1. Are there any major differences between Sweden and other countries in healthcare resource usage that could affect the generalizability of the findings?

Response: Thank you for your comment. There are differences in health care systems and principles for resource allocation between countries. The results of this study, and the relation between SRH and mortality, should not be largely affected by this. Healthcare use could potentially be affected depending on the system, social economic structure and access to in-hospital care. The results are considered to be generalisable between countries with similar socio-economic status and healthcare systems. This has been added to the discussion.

2. The term "EQ-VAS" should be fully spelled out the first time it is introduced.

Response: Thank you for pointing this out, we have now changed to EQ Visual Analogue Scale, p 5, line 173. It is provided by the EuroQoL group but is not spelled out in the instrument name.

3. Is it appropriate to group participants by SRH only once at the beginning of the study? Consider discussing potential changes in individual SRH responses during the two-year follow-up, particularly in this vulnerable population.

Response: In this study we have not studied differences over time, we have been interested in studying and describing the relation between different factors (SRH and mortality) for this population. However, we agree that it would be very interesting for further studies to also explore changes over time.

4. The data on hospital care days (p.7, lines 49–50) and physician office visits (p.7, line 57–p.8, line 10) would be clearer if presented in a table.

Response: Thank you for pointing this out, we agree. We have added a table (see table 3) and removed figure 4.

5. For Figures 2 and 3, please provide the "Number at risk." Also, consider including a table with hazard ratios (HRs) where appropriate.

Response: The number at risk has been provided, see figure 2 and 3.

6. On p.9, line 15, the finding that there is little correlation between SRH and mortality or hospital care days in older adults at high risk of hospitalization is a key result. However, the discussion lacks depth and relies too much on speculation based on previous studies. The manuscript should provide a more thorough explanation of why this finding emerged.

Response: Thank you for this comment. Due to other reviewer comments, we have expanded the analyses and the discussion to explain our findings. The new analyses led to an update of the results, please see Results, p 5, line 230-238 and Discussion, p 9, line 306-326.

7. On p.9, line 57, the manuscript suggests there is no reason to expect other items in the

questionnaire to significantly affect SRH perception. However, this statement requires further justification or supporting evidence.

Response: Thank you for pointing this out. We agree that this sentence was not well formulated. We have now removed this statement from the manuscript.

Reviewer: 2
Prof. Zoe Kelson, University of Exeter
Comments to the Author:
This study evaluated the predictive value of self-rated health (SRH) and the impact of comorbidities on morbidity and mortality in older adults (aged 75+) at high risk of hospitalisation.

Reviewer comments:

1. "There was no significant difference between the three groups of SRH in terms of mortality or hospital care days. However, groups with higher CCI had significantly higher mortality and a greater number of hospital care days." [Abstract]
Can statistical outcomes please be reported alongside study results in the Abstract?

Response: Thank you for this comment, we have updated the abstract to include statistical outcomes in the study results.

2. "The original trial included individuals from 19 primary care practices in Sweden, aged ≥75 years and at high risk of hospitalisation selected using a prediction model. A total of 1308 participants received a baseline questionnaire; 355 individuals were included." [Abstract]
and
"A total of 1308 participants were selected in March 2017 using a statistical prediction model that calculates a risk score for hospital admission in the coming 12 months using routine health care data from the preceding year...
The selected participants represented the 11% with highest risk score of the total population over 75 years at the 19 practices."
and
"The questionnaire was provided to a total number of 1308 participants. The survey was completed by 798 individuals, out of which 355 respondents consented to further analysis and had responded to the primary SRH-question"
Can the authors please confirm if the included cohort can be considered to be representative for generalisability of the study findings?

Response: Thank you for this comment. We have updated the sections Results and Strength and limitations. In comparison with the characteristics of the complete cohort of the intervention trial (See reference 42, Costs and effects of comprehensive geriatric assessment ....) our sub-population had a higher percentage of men, but no other significant differences. We consider that the results are generalisable to older adults at high risk of hospitalisation in countries with similar socio-economic status and healthcare systems. (Discussion line 370-382).

3. Can differences by primary care practice please be explored?

Response: Thank you for this comment. We have now commented primary care practices in the manuscript, p 4, line 150-152. The Swedish healthcare system is tax-funded and provides universal access to care for all residents, with services primarily managed by regional authorities. Primary care practices are comparable in terms of care.

4. "The questions covered background information (e.g., age, socioeconomic factors); need for assistance in daily life; loneliness; mental aspects; pain and health related quality of life (HRQoL) measured with the EQ-5D-3L (EQ-5D descriptive system three levels) and the EQ-VAS"
and
"Data were imported from the County Council of Östergötland computerised information system, where statistics for all healthcare in the county are stored (Care data warehouse, described in previous article)."
Can the authors please confirm if missing data (with covariate missing data shown in Table 1, and outcome missing data shown in Figure 1) can be considered to be missing at random?

How was covariate missing data handled in the analyses and models?

Response: Thank you for these comments. We adjusted all analyses for age, sex and level of education. We handled missing values of level of education as a separate group in our analysis to include all cases. (see Statistical analysis line 191-200)

5. "Self-rated health was assessed by the question:" In general, would you say your health is: Excellent, Very good, Good, Fair or Poor". The responses were divided into three groups: Good, Fair and Poor. "
and
"As few respondents rated their health as Excellent or Very good, groups were combined for further analysis into three categories: Good (including Excellent, Very good and Good); Fair (fair) and Poor (poor)."
Can the outcome please also be analysed as five groups in an exploratory sensitivity analysis?

Response: Thank you for this comment, it helped us to improve the manuscript. We have updated our analyses to four groups or SRH ratings instead of five, as the group Excellent included only n=2 participants. The analysis showed some changes in the results and affected our discussion slightly. A trend of a graded relationship between SRH and mortality and healthcare use emerged, however not statistically strong enough to conclude that SRH could be used for predictive purposes concerning mortality or healthcare use. We have updated Method, Results and Discussion with the new analyses and results.

6. "Differences in categorical variables were analysed using Chi-square test and continuous variables using one-way ANOVA. Cumulative mortality was performed using Kaplan-Meier and Cox regression was used to calculate hazard ratios (HRs) for mortality in the different groups of SRH and CCI, respectively. Comparisons of mean values of hospital care days and Physician visits were analysed with a one-way ANOVA grouped by SRH and CCI, separately. In case of a significance, a Tukey post hoc test was conducted."
Can the authors please confirm if and how they assessed normality for the application of parametric tests (e.g. ANOVA)?

Response: Thank you for this comment. We changed the analyses for differences in categorical variables to another parametric method, Fisher's exact test to better handle categories with few values. Continuous variables were still analysed using one-way ANOVA as we wanted to analyse mean values. However, we changed our analysis method for count data (Hospital care days and physician visits) and instead use negative binomial regression adjusted for sex, age and level of education. This method is more suitable for count data than for instance linear regression, even though we get almost the same results using linear regression.

7. Can the authors please clarify if the Cox regression analyses adjusted for confounders? Can covariates please be specified here in the methods?

Response: Thank you for this comment. We have clarified the analyses, with adjusted models for age, sex and level of education. See Table 2.

8. "the majority were men (56.9%), with no statistical difference between the groups of SRH" Can the authors please double check the wording in the Results text, as Table 1 shows there is a statistical difference in sex ratio between the three groups ($p<0.001$)?

Response: Thank you for pointing this error out. We have updated the Result section and Table 1.

9. "The three groups of SRH had significant differences ($p\leq0.001$) in EQ-VAS scores, with the highest average rating of EQ-VAS in SRH Good 67.7 (SD 19.2), followed by SRH Fair 51.6 (SD 16.7) and SRH Poor 34.0 (18.8). (Table 1) "
and
"Table 1. Characteristics of study participants, grouped by SRH"
Can statistically significant differences in sex, education and EQ-VAS score (shown in Table 1) please be commented on in the Results text, and adjusted for in the analyses?

Response: Thank you for this comment. We have updated the result section, see table 2 and 3, and p 7 line 219-225. EQ-VAS cannot be adjusted for as it has a strong correlation with the outcome SRH.

10. Figure 2:
Can the dichotomisation of 'Poor' versus 'not-Poor' (i.e. 'Fair' and 'Good' combined) SRH outcome please be analysed? It might be that the cohort analysed in this study is better at differentiating between 'Poor' and 'Fair and above' health statuses, than using more granular categories such as 'fair', 'good', 'very good' and 'excellent'?

Response: Thank you for this comment, we developed our manuscript by performing new analyses which led us to updated results. However, we chose not to dichotomise the health statuses, instead we grouped the SRH levels in four and performed new analyses using Poor as the reference. Excellent/Very good emerged as significantly different for both crude and adjusted HR compared to Poor, as well as adjusted Fair to Poor.

11. "The data set was relatively small compared to studies on SRH and correlation to mortality and hospitalisation, based on epidemiological data. However, our cohort was larger or of similar size compared to questionnaire-based studies on vulnerable adults and therefore add value to

the research field.23 The five categories of SRH had to be grouped together to compensate for small groups of the higher ratings of SRH. This is consistent with the methodology of similar studies, as the groups of higher ratings tend to be smaller and rarely shows significant differences in comparing analyses in the range from Good to Excellent.17, 34 As the SRH question is embedded in a larger questionnaire, other items might affect the individuals' perception of their health, i.e., question order effects. We did not investigate this aspect further in the present study, but we have no reason to believe that it would have a significant impact on the results."
A discussion on the study limitations has been provided.

Response: Thank you for this comment. The last sentence has been removed and the discussion on Strengths and limitations is updated.

12. Thanks for providing a copy of the STROBE checklist.

Response: The STROBE checklist has been updated after the revision.

Reviewer: 1
If you have selected 'Yes' above, please provide details of any competing interests.: Not applicable

Reviewer: 2
If you have selected 'Yes' above, please provide details of any competing interests.: Not applicable

---

VERSION 2 - REVIEW

**Reviewer**	**2**
**Name**	**Kelson, Zoe**
**Affiliation**	**University of Exeter, Mathematics**
**Date**	**14-Jul-2025**
**COI**	

Thanks to the authors for responding to each comment in turn, amending the analysis and revising the article.

Author response:

"We adjusted all analyses for age, sex and level of education. We handled missing values of level of education as a separate group in our analysis to include all cases. (see Statistical analysis line 191-200)"

Reviewer request:

Can the authors please clarify if missing data can be considered to be missing at random?

Author response:

"Continuous variables were still analysed using one-way ANOVA as we wanted to analyse mean values."

Reviewer request:

Can the authors please clarify if and how they checked for normality?

VERSION 2 - AUTHOR RESPONSE

Prof. Zoe Kelson, University of Exeter

Comments to the Author:

Thanks to the authors for responding to each comment in turn, amending the analysis and revising the article.

Author response:

"We adjusted all analyses for age, sex and level of education. We handled missing values of level of education as a separate group in our analysis to include all cases. (see Statistical analysis line 191-200)"

Reviewer request:

Can the authors please clarify if missing data can be considered to be missing at random?

Response: We understand that you want a more elaborate answer to this question. There is no definitive method to determine whether data are missing at random. However, as an attempt, we conducted four separate logistic regression analyses. In each model, the dependent variable represented missingness (coded as 1 for missing and 0 for non-missing) for the following variables: (1) level of education, (2) accommodation, (3) cohabitation status, and (4) EQ-VAS. The independent variables included age, sex, Charlson Comorbidity Index (CCI), and riskscore. None of the models yielded statistically significant associations between the explanatory variables and missingness. These findings suggest that missingness in these variables is not systematically related to age, sex, CCI, or riskscore, providing some support for the assumption that the data may be missing at random.

Author response:

"Continuous variables were still analysed using one-way ANOVA as we wanted to analyse mean values."

Reviewer request:

Can the authors please clarify if and how they checked for normality?

Response: Thank you for asking for a clarification: In the main analyses, where count data served as the dependent variables, we employed negative binomial regression to account for the skewed distribution. However, to assess differences in the means of continuous and discrete variables presented in Table 1, specifically age, EQ-VAS, and riskscore, we used one-way ANOVA. Given the sample sizes across the groups (nPoor = 49, nExcellent/Very good = 28, nGood = 90, and nFair = 188), the central limit theorem supports the assumption that sample means are likely representative of population means. As a sensitivity analysis, we also performed Kruskal-Wallis tests on these variables. The results were consistent with those from the ANOVA: age ($p = 0.27$ vs. 0.31), EQ-VAS ($p < 0.001$ in both tests), and risk score ($p = 0.72$ vs. 0.13). These findings suggest that the choice of statistical test did not substantially affect the interpretation of group differences. We have added a footnote to table 1 with the p-values from the Kruskal-Wallis tests.
